# Anaesthetic Management of One-Lung Ventilation in a Fontan Patient

**DOI:** 10.7759/cureus.89274

**Published:** 2025-08-03

**Authors:** Richard Coulie, Francis Ryckaert, Evgeny Raevsky, Victor Tsang, Susan Wright

**Affiliations:** 1 Department of Perioperative Medicine, Bart's Heart Centre, Barts Health NHS Trust, London, GBR; 2 Department of Cardiac Surgery, Bart's Heart Centre, Barts Health NHS Trust, London, GBR; 3 Department of Cardiac Surgery, Great Ormond Street Hospital, London, GBR; 4 Department of Perioperative Medicine, Bart’s Heart Centre, Barts Health NHS Trust, London, GBR

**Keywords:** fontan circulation, lateral thoracotomy, one-lung ventilation (olv), pacemaker lead repositioning, pulmonary vascular resistance

## Abstract

Patients with Fontan circulation are increasingly presenting for non-cardiac surgical procedures in adulthood, often involving complex anaesthetic management due to their unique physiology. We report the case of a 39-year-old Fontan patient who underwent elective thoracoscopic epicardial pacemaker lead revision under one-lung ventilation, requiring advanced hemodynamic monitoring, inotropic support, and pulmonary vasodilators. This case highlights the significant perioperative challenges in such patients and the need for a specialised, multidisciplinary team. In addition to this case, we reviewed previously published reports on one-lung ventilation in Fontan patients to provide a broader perspective on perioperative strategies and outcomes in this high-risk population.

## Introduction

Since 1968, tens of thousands of patients with anatomical or functional single ventricle physiology have undergone Fontan surgery as palliative treatment [1]. It is currently estimated that more than 70,000 individuals with a Fontan circulation are alive, and this number is expected to rise significantly in the coming decades [2]. Thanks to major advances in surgical techniques and medical management, the estimated 30-year survival rate now approaches 85%. Anaesthetists are increasingly likely to encounter these patients for a range of elective or urgent surgical procedures. Among the most common are interventions related to cardiac rhythm management such as cardioversion, epicardial pacemaker implantation (e.g. for sinus node dysfunction or atrioventricular block), or arrhythmia ablation (e.g. atrial tachyarrhythmia) [3]. Regardless of the clinical context, a thorough understanding of the unique physiology associated with Fontan circulation and the related systemic organ dysfunction, along with the key anaesthetic principles it entails, is essential for safe and effective care.

In a Fontan circulation, blood bypasses the subpulmonary ventricle and flows in a non-pulsatile manner from the systemic venous return across the pulmonary vascular bed to the common atrium. The maintenance of this flow depends on an adequate transpulmonary pressure gradient (TPPG) and low pulmonary vascular resistance (PVR). In a well-functioning Fontan circulation, central venous pressure (CVP), also known as "Fontan pressure", is only mildly elevated, rarely exceeding 18-20 cmH₂O. An increase in PVR leads to a reduction in pulmonary blood flow, which decreases systemic ventricular preload and subsequently lowers cardiac output (CO). When PVR is elevated, the presence of a fenestration (a connection between the caval system and the common atrium) may help augment preload and cardiac output and decrease systemic venous hypertension by allowing blood to bypass the pulmonary vasculature, though this comes at the cost of decreased systemic arterial oxygen saturation. The presence of a fenestration is highly variable, as there remains a wide variation in surgical practice, criteria and timing for fenestration due to insufficient evidence-based consensus.

One-lung ventilation (OLV) in Fontan patients undergoing surgery represents a particularly high-risk challenge. Hypoxia and hypercarbia can acutely increase PVR, reduce TPPG, and consequently decrease CO. Moreover, in patients with a fenestration, elevated PVR may further exacerbate right-to-left shunting through the fenestration, resulting in worsened hypoxemia. Only a limited number of case reports have described anaesthetic management in this context with various approaches and favourable outcomes [4-11].

In this case report, we present our management approach, review the existing literature, and summarise key anaesthetic considerations for performing OLV in patients with Fontan physiology.

## Case presentation

We describe a 39-year-old male Fontan patient scheduled for thoracotomy under general anaesthesia for replacement of atrial and ventricular epicardial pacing leads, along with a pacing box exchange due to rising pacing thresholds.

His primary cardiac diagnosis was tricuspid atresia. At the age of three, he underwent creation of an atrio-pulmonary connection (classical Fontan), with closure of an atrial septal defect (ASD). During adolescence, he developed paroxysmal atrial fibrillation and at the age of 23, he underwent Fontan conversion with an extra-cardiac conduit (ECC), bilateral bidirectional cavo-pulmonary shunts, surgical Maze ablation, and implantation of a permanent dual-chamber epicardial pacemaker. The right superior vena cava (R-SVC) was connected to the right pulmonary artery (R-PA), and the left SVC to the left PA. At the age of 31, he underwent atrial flutter ablation. His epicardial pacemaker box was replaced one year later due to battery depletion.

Apart from a congenitally absent left kidney, he had no other significant medical history. His regular medications included sotalol 80 mg daily, perindopril 4 mg daily, and warfarin. The latter was discontinued five days before surgery, and his international normalised ratio (INR) was within the normal range on the day of the procedure.

Preoperative vital signs were within normal limits: non-invasive blood pressure (NIBP) 120/65 mmHg, heart rate (HR) 57 bpm, and oxygen saturation (SpO₂) 93% on room air. Although mild hypoxaemia is common in Fontan patients, his saturation was lower than expected. A preoperative computed tomography (CT) scan did not reveal any veno-venous collaterals or pulmonary arteriovenous malformations that could account for this finding, and no fenestration was present in the ECC repair.

Other than being overweight (weight 97 kg, height 180 cm, BMI 29.9 kg/m²), the clinical examination was unremarkable. He was a non-smoker and consumed alcohol occasionally. Preoperative blood tests were within normal limits, except for mildly abnormal liver function tests (Table 1).

**Table 1:** Preoperative laboratory values Hb: Haemoglobin. WCC: White cell count. INR: International normalised ratio. eGFR: Estimated glomerular filtration rate. ALT: Alanine transaminase. AST: Aspartate aminotransferase. Alk Ph: Alkaline phosphatase. CRP: C-reactive protein.

		Reference range
Hb	167 g L^-1^	140 - 180 g L^-1^
WCC	4.7.10^9^ L^-1^	4.5 - 11.10^9^ L^-1^
Platelet count	166.10^9^ L^-1^	150 - 350.10^9^ L^-1^
INR	1.2	0.9 - 1.1
Na^+^	142 mmol L^-1^	136 - 144 mmol L^-1^
K^+^	4.6 mmol L^-1^	3.5 - 5.5 mmol L^-1^
Cl^-^	98 mmol L^-1^	96 - 106 mmol L^-1^
Mg^2+^	0.9 mmol L^-1^	0.9 - 1.8 mmol L^-1^
Urea	6.8 mmol L^-1^	8 - 23 mmol L^-1^
Creatinine	1.01 mg dL^-1^	0.6 - 1.2 mg dL^-1^
eGFR	81 mL min^-1^ 1.73m^-2^	75 - 125 mL min^-1^ 1.73m^-2^
Total bilirubin	1.28 mg dL^-1^	0.4 - 1.3 mg dL^-1^
ALT	43 U L^-1^	10 - 38 U L^-1^
AST	41 U L^-1^	10 - 36 U L^-1^
Alk Ph	102 U L^-1^	30 - 120 U L^-1^
CRP	2 mg L^-1^	0.08 – 3 mg L^-1^

Preoperative transthoracic echocardiography (TTE) showed a well-functioning, non-fenestrated ECC repair with laminar, phasic flow in both superior and inferior vena caval pathways, and a non-restrictive ASD. The systemic ventricle had preserved radial and longitudinal function, with an estimated ejection fraction of 55%. Mild left atrioventricular valve regurgitation was observed, with a normal aortic valve and root.

The preoperative electrocardiogram (ECG) showed an atrially paced rhythm, QRS duration of 123 ms, global T-wave inversion, and a corrected QT interval of 412 ms. Underlying atrial standstill was noted. The pacemaker was set to DDD mode, with 100% atrial pacing and preserved atrioventricular conduction with ventricular sensing. Device interrogation revealed increasing impedances and thresholds in both atrial and ventricular epicardial leads, without any battery concerns.

During the preoperative team briefing, surgical and anaesthetic strategies were discussed, along with contingency plans for failed one-lung ventilation or haemodynamic instability. A stepwise approach was planned: initial medical optimisation to enhance pulmonary blood flow and cardiac output, followed, if needed, by return to two-lung ventilation. In the event of significant haemodynamic compromise, emergency cardiopulmonary bypass would be initiated via femoral arterial and venous cannulation, with a perfusionist present throughout the procedure.

To minimise fasting time, the case was scheduled in the morning. A 20-gauge peripheral venous cannula was inserted, and volume preloading with 500 mL Hartmann’s solution was initiated. Standard American Society of Anesthesiologists (ASA) monitoring was applied, including continuous invasive arterial blood pressure via right radial artery, bilateral cerebral near-infrared spectroscopy (NIRS), and processed electroencephalogram (pEEG). Self-adhesive defibrillator pads were applied pre-induction. Pre-induction arterial blood gas (ABG) on room air showed acceptable gas exchange and acid-base balance (Table 2).

**Table 2:** Intraoperative arterial blood gas and central venous pressure values OLV: One lung ventilation. Hb: Haemoglobin. Lac: Lactate. CVP: Central venous pressure (Fontan pressure).

	Pre induction	Post induction	OLV	Post OLV	Reference range
pH	7.42	7.32	7.30	7.32	7.35 - 7.45
PaCO_2_ (kPa)	5.0	6.6	6.9	6.5	4.6 - 5.9 kPa
PaO_2_ (kPa)	8.6	15.8	10.2	15.4	> 10 kPa
SaO_2_ (%)	94.1	98.7	94.6	98.6	> 90%
Hb (g L^-1^)	171	156	147	160	140 - 180 g L^-1^
Na^+^ (mmol L^-1^)	141	140	139	137	136 - 144 mmol L^-1^
K^+^ (mmol L^-1^)	4.3	4.5	5.1	5.5	3.5 - 5.5 mmol L^-1^
Cl^-^ (mmol L^-1^)	108	108	108	107	96 - 106 mmol L^-1^
Glucose (mmol L^-1^)	5.6	5.4	5.7	6.6	4.0 - 5.5 mmol L^-1^
Lac (mmol L^-1^)	0.6	0.7	0.9	0.7	< 2 mmol L^-1^
Base Excess (mmol L^-1^)	0.6	-0.9	-1.7	-1.8	-4 – 2 mmol L^-1^
HCO_3_^-^ (mmol L^-1^)	24.7	25.9	25.6	25.1	22 - 26 mmol L^-1^
CVP (mmHg)	-	25	18 - 22	19 - 23	-

The patient was preoxygenated with 100% FiO₂ for five minutes before induction. Pre-induction vitals were stable: HR 55 bpm, BP 135/65 mmHg, SpO₂ 100%. Anaesthesia was induced using remifentanil target-controlled infusion (TCI, Minto model, effect-site target 4 ng/mL) and a bolus of 150 mg propofol. Following loss of consciousness, a Guedel airway was placed to minimise airway pressure during gentle bag-mask ventilation, and rocuronium 0.6 mg/kg was administered.

Intubation was performed with a 39Fr left-sided double-lumen tube (DLT). After confirming tube placement, pressure-regulated volume control ventilation was initiated. The settings were: FiO₂ 100%, tidal volume (VT) 450 mL, respiratory rate (RR) 14/min, inspiratory-to-expiratory (I:E) ratio 1:3, and positive end-expiratory pressure (PEEP) 5 cmH₂O. Mean airway pressure (Paw) during two-lung ventilation reached 20 cmH₂O. Anaesthesia was maintained with remifentanil TCI and sevoflurane, titrated according to EEG and end-tidal agent concentrations.

Additional access was obtained post-induction via a 14-G peripheral cannula and a four-lumen central venous catheter in the right internal jugular vein. Fontan pressure was measured at 25 mmHg (Table 2). Intravenous fluids were connected to a fluid warmer. A transoesophageal echocardiography (TOE) probe and an oesophageal temperature probe were placed.

FiO₂ remained at 100% throughout the procedure. Flucloxacillin and gentamicin were administered for antibiotic prophylaxis as per local guidelines.

After an uneventful induction, the left lung was deflated, and haemodynamic and respiratory parameters were monitored during a 10-minute trial of one-lung ventilation (OLV) in our pre-induction room. As all parameters remained stable, two-lung ventilation was resumed in order to avoid unnecessarily prolonging the duration of OLV during patient transfer to the operating theatre, positioning and surgical preparations prior to incision. The pacemaker was reprogrammed to DOO mode 70 bpm.

He was positioned in a modified right lateral decubitus position, allowing access to the left hemithorax and midline abdomen. Pressure points were protected, and eyes taped. A low-dose glyceryl trinitrate (GTN) infusion (1 mg/h) was started for pulmonary vasodilation. Despite preloading, mean arterial pressure (MAP) dropped to 45 mmHg, prompting initiation of a vasopressin infusion (0.03 U/min). Metaraminol boluses (250-500 µg) were administered to maintain MAP above 65 mmHg.

**Figure 1:**
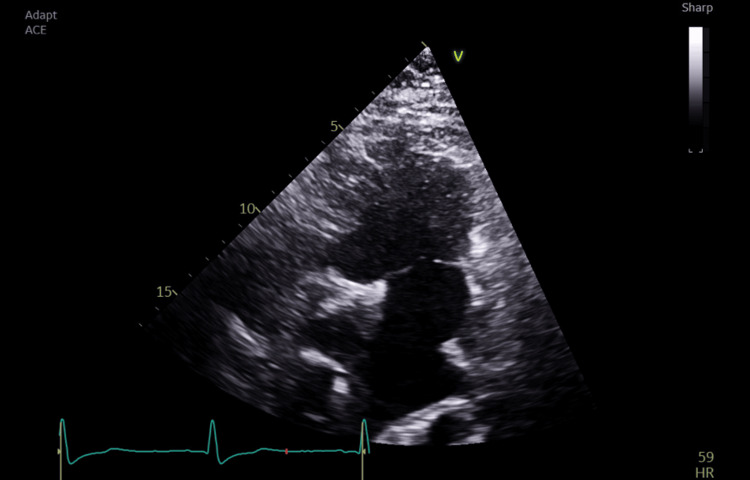
Deep transgastric five-chamber view showing the systemic ventricle with the left atrioventricular valve

**Figure 2:**
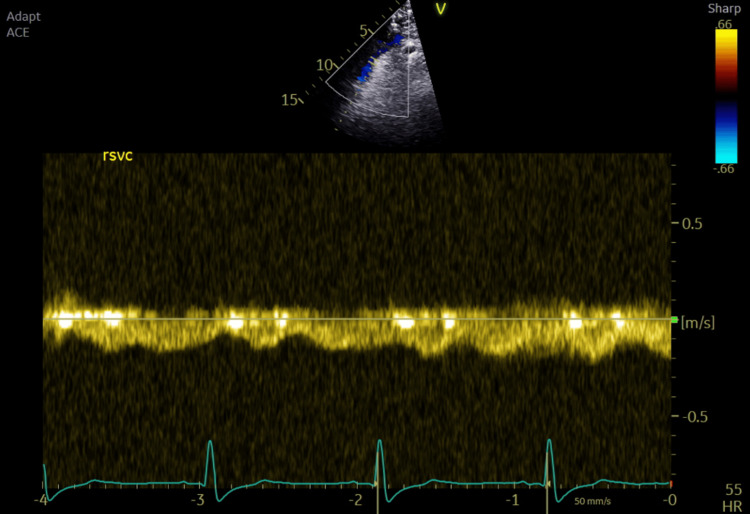
Velocities in the extra-cardiac conduit showing laminar flow with positive pressure ventilation variations

Intraoperative TOE showed good systemic ventricular function and left atrioventricular valve regurgitation. The total cavopulmonary connection (TCPC) demonstrated laminar flow in both caval pathways, with blunting and cessation of forward flow during positive pressure inspiration (Figures 1-2).

A left thoracotomy was performed via an incision between the fifth and sixth ribs. OLV was re-initiated by deflating the left lung (VT 400 mL, RR 15/min, I:E ratio 1:3, PEEP 5 cmH₂O, Paw 29 cmH₂O) to improve surgical exposure. Continuous positive airway pressure (CPAP) of 5 cmH₂O was applied to the non-ventilated lung. Vasopressin was increased to 0.04 U/min, and inhaled nitric oxide (iNO) was started prophylactically at 20 ppm. OLV was maintained for over 25 minutes with stable MAP and SpO₂ (see Table 2 for ABG values). End-tidal CO₂ ranged from 5.0 to 5.2 kPa (normal 4.6-5.9); PaCO₂ peaked at 6.9 kPa.

Surgical dissection was difficult due to previous interventions and distorted anatomy. Twenty-nine minutes after initiating OLV, ventricular fibrillation (VF) occurred due to accidental electrocautery contact with the left ventricle. Four direct-current shocks (360 J each) and 300 mg of amiodarone were required to restore paced rhythm after about 2 minutes. Prophylactic lidocaine 100 mg and magnesium sulfate 5 g were administered. During the event, BP dropped to 74/56 mmHg and SpO₂ to 94%, prompting a return to two-lung ventilation. Minute ventilation was increased, and iNO was titrated to 40 ppm. TOE showed mild-to-moderate left ventricular dysfunction. Dobutamine was initiated at 5 µg/kg/min, without the need for an increased rate.

Following 15 minutes of stabilisation, surgery resumed. As the atrial lead had acceptable thresholds and the left atrium was inaccessible with two-lung ventilation, it was left in place. A new ventricular epicardial lead was placed without returning to OLV. The pacing box was replaced via a mini midline laparotomy. After tunnelling the ventricular lead, the new system was functional with low thresholds for both leads.

Dobutamine, vasopressin, GTN, and iNO were gradually weaned at the end of surgery, with TOE assessment confirming restoration of systemic ventricular function to the preoperative baseline. Multimodal analgesia was administered: levobupivacaine infiltration, intravenous paracetamol 1 g, ketamine 0.15 mg/kg, slow infusion of clonidine 150 µg, and fentanyl PCA (10 µg bolus, 5-minute lockout). Thoracic epidural was not offered in light of the risk of hypotension and the need for therapeutic anticoagulation. Given that the patient's hemodynamic and respiratory status were excellent and remained stable over time, with no further need for inotropic or vasoactive drug support and normal gas exchange, a decision was made to wake him up in the operating theatre. Spontaneous ventilation would even improve the patient's status. The DLT was exchanged for a supraglottic airway (i-gel®, Intersurgical, Berkshire, UK) to allow spontaneous ventilation. Neuromuscular blockade was reversed with sugammadex, and postoperative nausea and vomiting prophylaxis was given (ondansetron 4 mg and dexamethasone 3.3 mg). The patient was extubated in the theatre without cardiovascular support. Blood loss was minimal. No blood products transfusion was needed. A total of 3500 mL Hartmann’s solution was administered intraoperatively. The total duration of surgery was approximately 110 minutes, with a total OLV time of 31 minutes.

Postoperative recovery in the ICU was uneventful. Neurological assessment revealed no abnormalities. Mild hypoxaemia (8 kPa) and hypercapnia (6.2 kPa) were managed with high-flow nasal oxygen for six hours, followed by nasal cannulae. Chest drains were removed on postoperative day 1, and the patient was transferred to the cardiac ward. He was treated empirically with amoxicillin-clavulanic acid for five days for suspected pneumonia and discharged home on postoperative day 5. Warfarin was restarted on POD 2.

## Discussion

To our knowledge, this is only the eighth case report discussing the management of one-lung ventilation in a Fontan patient. Anaesthetic management described in the previous case reports is summarised in Table 3, and we elaborate on the techniques here.

**Table 3:** Summary of previously published case reports A systematic search of published studies and case reports was conducted on PubMed using the terms ("Fontan") and ("One lung ventilation"). Seven case reports were identified and included in our review. One case series by Haight et al. was not included as the anaesthetic management is not detailed [11]. OLV: One-lung ventilation. Intraop: Intraoperative. BMI: Body mass index. TCPC: Total cavopulmonary connection. LTC: Lateral tunnel cavopulmonary connection. IBP: Invasive blood pressure. NIBP: Non-invasive blood pressure. CVP: Central venous pressure. rSO2: Regional cerebral oxygen saturation. CPB: Cardiopulmonary bypass. ETT: Endotracheal tube. BB: Bronchial blocker. VT: Tidal volume. RR: Respiratory rate. Paw: Peak inspiratory pressure. n/a: No data available. PMH: Past medical history. ECG: Electrocardiogram. DLT: Double lumen tube. VC: Volume-controlled ventilation. PC: Pressure-controlled ventilation. PRVC: Pressure-regulated volume-controlled ventilation. PEEP: Positive end-expiratory pressure. iNO: Inhaled nitric oxide. ICD: Implanted cardioverter-defibrillator. RIJV: Right internal jugular vein. PRBC: Packed red blood cells. U: Unit. FFP: Fresh frozen plasma. Plat: Platelets. TCI: Target-controlled infusion. PCEA: Patient-controlled epidural analgesia. TOE: Transoesophageal echocardiogram. ECMO: Extracorporeal membrane oxygenation. TIVA: Total intravenous anaesthesia. VF: Ventricular fibrillation.

	Majima et al. 2012 [4]	Cvetkovic et al. 2014 [5]	Gregory et al. 2016 [6]	Sasaki et al. 2019 [7]	Higashi et al. 2024 [8]	Kawakami et al. 2024 [9]	Hibino et al. 2024 [10]	This paper
PATIENT CHARACTERISTICS								
Age (years)	12	28	41	41	18	16	28	39
Sex	Female	Male	Male	Female	Male	Male	Female	Male
Weight (kg)	47	83	n/a	n/a	45	69	61	97
Height (cm)	152	198	n/a	n/a	163	169	160	180
BMI (kg/m^2^)	20.3	21.1	n/a	n/a	17.2	24	23.8	29.9
Congenital heart disease	Single atrium, Single ventricle, Pulmonary stenosis, Persistent left superior vena cava	Hypoplastic left heart syndrome	Tricuspid atresia	Single ventricle type defect, Hypoplastic right ventricle	Hypoplastic right heart, Tricuspid stenosis, Pulmonary stenosis, Patent ductus arteriosus, Foramen ovale	Dextrocardia, Double inlet right ventricle, Double outlet right ventricle, Modified great artery transposition	Tricuspid atresia	Tricuspid atresia
Fontan type	TCPC	Non-fenestrated LTC	Non-fenestrated TCPC	TCPC	n/a	TCPC	Non-fenestrated TCPC	Non-fenestrated TCPC
Other PMH	n/a	n/a	Maze procedure for atrial fibrillation, Abdominal implanted ICD	Atrial flutter, Permanent pacemaker, Left hemidiaphragmatic paralysis, Menorrhoea, Thrombocytopenia	n/a	Permanent pacemaker for sick sinus syndrome	Sick sinus syndrome, Paroxysmal supraventricular tachycardia, Cirrhosis (Child-Pugh B)	Atrial flutter, Permanent epicardial pacemaker, Absent left kidney
Medications	n/a	Enalapril, Clopidogrel, Budesonide/Formoterol	Benazepril, Carvedilol, Torsemide, Spironolactone, Warfarin	Diuretics	Aspirin, Warfarin	n/a	n/a	Sotalol, Perindopril, Warfarin
Ventricular ejection fraction	Preserved	Preserved	Preserved	Preserved	n/a	Preserved	Preserved	Preserved
Room air SpO_2_	85%	92 - 94%	94% (FiO_2_ 35%)	n/a	n/a	n/a	92	93%
Preoperative CVP (mmHg)	11	n/a	20	16	n/a	14	8	n/a
Planned surgery	Elective right open thoracotomy for treating varices from the right superior vena cava to the right pulmonary vein (risk of rupture and thrombus)	Elective left open thoracotomy for lung biopsy (left lower lobe nodule)	Urgent left open thoracotomy for decortication (loculated left-sided pleural effusion context of streptococcal toxic shock syndrome)	Semi-elective left open thoracotomy for left diaphragmatic plication	Elective right thoracoscopic pulmonary cystectomy (pneumothorax)	Elective left thoracotomy for epicardial atrial pacemaker lead implantation	Elective right-sided minimally invasive thoracotomy for epicardial pacemaker lead implantation	Elective left open thoracotomy for epicardial pacemaker lead and box change
INTRAOPERATIVE DATA								
Anaesthetic monitoring	Basic monitoring (?), IBP, CVP (right superior vena cava), rSO_2_	Pulse oximeter, ECG, NIBP + IBP	Standard monitoring (?), IBP, CVP (RIJV)	IBP, CVP (RIJV), TOE	IBP, CVP (RIJV), TOE	IBP, CVP, TOE	Pulse oximeter, ECG, IBP, CVP, TOE	Pulse oximeter, ECG, IBP, CVP (RIJV), EEG, Sedline, rSO_2, _TOE, Defibrillator patches
Back-up	CPB	iNO	iNO	iNO, CPB	n/a	iNO, ECMO	n/a	ECMO
Intraoperative pacing mode	-	-	-	DOO 70bpm	-	AAI 80bpm	-	DOO 70bpm
Induction drugs	Sevoflurane, Vecuronium	Midazolam 2mg, Fentanyl 100µg, Etomidate 20mg, Rocuronium 50mg	Fentanyl, Lidocaine, Etomidate, Succinylcholine	Propofol TCI 2.5µg mL^-1, ^Fentanyl 100µg, Rocuronium	Fentanyl 100µg, Propofol TCI 3µg mL^-1, ^Remifentanil 0.2µg kg^-1^min^-1^	Propofol TCI 2.2-2.5µg mL^-1, ^Remifentanil 0.2-0.4 µg kg^-1^ min^-1^	Midazolam, Ketamine, Remifentanil, Rocuronium	Propofol 150mg, Remifentanil TCI 4ng mL^-1, ^Rocuronium 70mg
Maintenance drugs	Sevoflurane, Fentanyl (2µg kg^-1^ h^-1^), Vecuronium	Sevoflurane	Desflurane, Fentanyl, Cisatracurium	Propofol TCI, Remifentanil, Rocuronium	Propofol TCI, Remifentanil	Propofol TCI, Remifentanil	Sevoflurane, Remifentanil	Sevoflurane, Remifentanil TCI, Rocuronium
Vasoconstrictor/vasodilator & inotropic drugs	Nitroglycerin, Dobutamine 3µg kg^-1^min^-1, ^Dopamine 5µg kg^-1^ min^-1^	None	Dopamine (preop), Milrinone (preop), Esmolol, Phenylephrine boluses, Vasopressin boluses	Ephedrine bolus	Dobutamine	Dobutamine 2-3µg kg^-1^min^-1, ^Norepinephrine 0.01-0.05µg kg^-1^ min^-1^	Dopamine 2.0-2.4µg kg^-1^ min^-1^, Dobutamine 2.0-2.4µg kg^-1^ min^-1^, Milrinone 0.16µg kg^-1^min^-1^,Nitroglycerine 0.14µg kg^-1^ min^-1^	Glyceryl Trinitrate 1mg/h, Metaraminol boluses, Vasopressin 0.02-0.04U min^-1^,Dobutamine 5µg kg^-1^min^-1^, iNO 20-40ppm
Crystalloid volume infused	2040mL (43mL kg^-1^)	1600mL (19mL kg^-1^)	1500mL	n/a	1575mL (35mL kg^-1^)	n/a	n/a	3500mL (36mL kg^-1^)
Colloid volume infused	None	None	500mL	n/a	None	n/a	n/a	None
Blood products given	None	None	PRBC 2U, FFP 1U, Plat 1U	n/a	None	n/a	n/a	None
Intraoperative complications	None	None	Surgical bleeding (1.5L), Hemodynamic instability	None	Non-expansion of the right lung requiring post-op negative pressure via a thoracic drain	None	None	VF
Analgesia	Fentanyl 1µg kg^-1^ h^-1^	n/a	n/a	PCEA Levobupivacaine	PCA	Fentanyl 50µg h^-1^,Intercostal nerve block, Levobupivacaine 0.25% 5mL h^-1^	n/a	Paracetamol, Ketamine, Clonidine, PCA Fentanyl
ONE-LUNG VENTILATION PARAMETERS								
Tube	ETT + BB 7Fr	Left-sided DLT 37Fr	Left-sided DLT 39Fr	Left-sided DLT 35Fr	Left-sided DLT 35Fr	DLT 32Fr	Left-sided DLT 35Fr	Left-sided DLT 39Fr
FiO_2_ (%)	100	100	100	100	n/a	100	100	100
SpO_2_ (%)	> 83	94 - 95	> 90	> 95	n/a	n/a	> 83	> 94
Ventilation mode	VC	PC	PC	PC	n/a	PC	PC	PRVC
Tidal volume (mL)	320	n/a	n/a	n/a	n/a	350 - 400	300	400
Respiratory rate (/min)	15	16 - 18	n/a	n/a	n/a	12 - 15	14	15
I/E ratio	n/a	1:2.5	1:3 - 1:4	1:2.6	n/a	n/a	n/a	1:3
Paw (cmH_2_O)	34	30	25 - 30	22	n/a	30	24	29
PEEP (cmH_2_O)	4	0	2 - 4	5	n/a	5	0	5
EtCO_2_ (mmHg)	< 40	30	30 - 35	< 40	n/a	< 40	n/a	< 40
PEEP on the excluded lung	n/a	n/a	2L/min O_2_	n/a	n/a	n/a	2 - 8L/min O_2_	5cmH_2_O
OLV duration (min)	230	75	n/a	85	n/a	About 220	About 250	31
POSTOPERATIVE DATA								
Extubation	In theatre	In theatre	In the intensive care unit	In theatre	In theatre	In theatre	In theatre	In theatre
Postoperative stay	n/a	Intensive care unit	Intensive care unit	n/a	Intensive care unit	Intensive care unit	Intensive care unit	Intensive care unit
Postoperative complications	n/a	None	Anasarca	None	None	None	None	Hypoxemia, hypercapnia, Suspicion of pneumonia

Although all of the cases had undergone Fontan palliative surgery, there is significant heterogeneity in the underlying congenital heart disease diagnoses, associated comorbidities, preoperative clinical status, and surgical procedures. While the indication for OLV was thoracotomy for all, the specific indications for surgery varied, making it challenging to classify this rare population as a single, homogeneous group for OLV management. Such variability may contribute to the differing anaesthetic management strategies, despite a shared focus on lowering PVR using a range of ventilatory settings and pharmacological agents tailored to individual patient profiles.

The case-report-based nature of this review limits the ability to establish a standardised approach suitable for all patients with Fontan physiology, yet it provides insight into various effective strategies used in specific clinical scenarios.

Preoperative assessment and eligibility

A thorough understanding of the patient’s physiology and previous cardiac surgeries is essential to adequately assess perioperative risks in patients with adult congenital heart disease (ACHD). Cardiac assessment, with at least a recent ECG and TTE performed by an experienced ACHD team, has become the standard of care. As patients with Fontan circulation are known to have multi-organ morbidity, preoperative investigation of all vital organs (including the lungs, liver and kidneys) is necessary. Depending on the type of surgery, various risk assessment tools may be available. If the procedure is considered medium or high risk, the patient should be treated in a specialised ACHD centre [12]. However, there are currently no objective risk assessment tools capable of accurately quantifying perioperative risk in ACHD patients undergoing non-cardiac surgery. This is largely due to the significant heterogeneity of this population, combined with their relative rarity. Such limitations are even more pronounced in the context of procedures involving OLV. Future prospective registries or ACHD databases could help fill the evidence gap in surgical risk assessment.

Intraoperative management

The primary goal in the anaesthetic management of a Fontan patient is the maintenance of adequate pulmonary blood flow, and thus, oxygenation and cardiac output. As the Fontan pressure gradient and PVR are the major determinants of cardiac output in single-ventricle physiology, hypovolaemia should be avoided at all costs, and all possible measures to keep PVR low should be prioritised.

Ventilatory Management

Positive pressure ventilation during general anaesthesia presents a particular challenge in Fontan patients and should focus on minimising PVR. It is well-documented that, in these patients, blood flow through the lungs ceases during positive pressure inspiration, as the increase in intrathoracic pressure during inspiration impedes forward blood flow from the systemic venous system.

This leads to a decrease in cardiac output [13]. To maximise cardiac output, forward flow through the lungs can be promoted by allowing for a longer expiratory time, at the expense of higher inflation pressures. There is, nevertheless, no consensus on which I/E ratio to use in such cases; instead, one should focus on maintaining adequate cardiac output. We used an I/E ratio of 1:3, which was well-tolerated. Other published case reports also use an I/E ratio ranging from 1:2.5 to 1:4.

Although PEEP and CPAP cause a rise in PVR, low levels of positive pressure applied to ventilated or non-ventilated lungs might be beneficial by preventing atelectasis-induced elevation of PVR. Patients with impaired spontaneous breathing, such as phrenic nerve paralysis, may have their CO improved by positive pressure ventilation by decreasing atelectasis. Five out of eight case reports mention the use of PEEP/CPAP during surgery.

Both hypoxia and hypercapnia increase PVR. The intrapulmonary shunt created during OLV compromises gas exchange. Any resulting hypoxia increases PVR by inducing hypoxic pulmonary vasoconstriction (HPV), which begins within the first few seconds of OLV, peaks after about two hours [14], and persists for hours into the postoperative period despite restoration of normoxia. Oxygen is a powerful pulmonary vasodilator, and a high FiO₂ is recommended to maintain a PaO₂ greater than 10 kPa. All other pharmacological measures to blunt this hypoxic pulmonary vasoconstriction should be considered (see PVR management). While a high FiO₂ does increase the risk of lung injury due to a pro-inflammatory response and the generation of reactive oxygen species, it seems common practice in all published literature to keep FiO₂ maximal intraoperatively, as the PVR-blunting effect is considered more important than potential postoperative inflammatory injury.

Even a modest degree of hypercarbia increases PVR, although its effect is slow-acting [15]. Mild hyperventilation, targeting a PaCO₂ of 4.0-4.6 kPa and thus avoiding respiratory acidosis, seems reasonable. While we did not manage to achieve this target, our end-tidal CO₂ remained within normal ranges. Kawakami and colleagues described a PaCO₂ of up to 7.3 kPa (55 mmHg), with an EtCO₂ of 4.8 kPa (36 mmHg) during the OLV period. An increasing arterio-alveolar gradient may be a sign of decreased pulmonary circulation and should be addressed promptly.

If expertise is available, selective blocking of a single lobe (e.g. only the left upper lobe to increase visualisation of the surgical field for epicardial pacemaker implantation) may benefit haemodynamics by limiting both the area impacted by HPV and the extent of lung atelectasis.

Fluid Management

Cardiac output in Fontan patients is heavily preload-dependent, and avoiding hypovolaemia is essential. Reduction of preoperative fasting times and administration of intravenous fluids during the period of starvation help to preserve an acceptable fluid status. Drinking clear fluids should be encouraged up to two hours before surgery [16]. Fasting times should be adapted when Fontan-associated cirrhosis with ascites or protein-losing enteropathy is present, as standard protocols may not be appropriate in these cases.

Peri-induction fluid loading helps to maintain cardiac output during this haemodynamically volatile period. Likewise, intraoperative hypotension should prompt administration of a fluid bolus before vasoconstrictors are given. Four out of eight published case reports indicate having administered large amounts of fluids (at least 1500 mL) intraoperatively [4-6,8]. This represents more than 35 mL/kg for 3 out of those 4 patients [4,8].

CVP measurement, although imperfect as a monitor of intravascular volume, is a useful surrogate for fluid status and preload. If available, TOE can be a helpful tool to assess filling. While the deleterious effects of hypovolaemia are widely recognised, it is worth noting that fluid overload can also be harmful in Fontan patients, as it increases the risk of atrial arrhythmias, and pulmonary oedema will elevate PVR.

Six out of eight reported cases used intraoperative CVP monitoring [4,6-10]. The use of TOE was described in only four of the previously published cases [7-10].

Pharmacological PVR Management

As an increase in PVR directly lowers the amount of blood flow reaching the systemic ventricle, it is imperative to avoid PVR-raising triggers and rises in PVR should be treated promptly. Pharmacologically, PVR can be decreased by multiple agents.

The choice of anaesthetic maintenance drug is complex in OLV in a Fontan patient. Published work indicates that there is no difference in arterial oxygen saturation (SaO₂) or systemic oxygen delivery (DO₂) between administering propofol or sevoflurane during OLV [17-19]. Nonetheless, sevoflurane, like all volatile agents, is known to partially inhibit HPV [20], in contrast to propofol. Maintenance of anaesthesia with sevoflurane could, at least in theory, limit the PVR increase induced by OLV but may contribute to a degree of hypoxia induced by HPV inhibition.

Low-dose GTN effectively lowers PVR [21-24]. Three other case reports [4,6,10] described the safe use of GTN. The use of GTN remains controversial, as it simultaneously reduces systemic vascular resistance, necessitating the use of vasopressors.

Inhaled nitric oxide (iNO), a potent selective inhaled pulmonary vasodilator, can be used either pre-emptively or as rescue therapy, as described by four other teams [5-7,9]. Nonetheless, care must be taken with iNO therapy, as rebound pulmonary hypertension may occur, especially after prolonged administration [25]. We chose to use it early to maximise pulmonary blood flow during OLV, selectively vasodilating well-ventilated areas of the lung and thus improving the ventilation/perfusion ratio. It was discontinued at the end of surgery without any signs of rebound PHT.

If vasoconstrictors are needed, vasopressin should be the agent of choice due to its limited effect on the pulmonary vasculature compared to adrenergic agents such as noradrenaline [23,26-27], which induce significant increases in PVR. Metaraminol, an α₁-adrenergic receptor agonist with some β-adrenergic effect, can be used safely as a bolus drug [26], as can phenylephrine [6] and ephedrine [7].

To reduce an increase in PVR induced by nociception or pain, a multimodal analgesia approach is appropriate. Short-acting, powerful opioids, such as remifentanil, can be used to provide adequate blunting of nociception during surgery and allow spontaneous ventilation and smooth emergence from anaesthesia at the end of surgery. Analgesic doses of both clonidine and ketamine can be used safely for pain management without increasing PVR [28], even though they have not been studied in the Fontan population [29] and can alter the patient's hemodynamic status. Infiltration of the wounds with long-acting local anaesthetic agents decreases the need for supplemental opioid analgesia, thereby reducing the risk of hypoventilation with resulting hypoxia and hypercapnia.

There is a place for patient-controlled epidural analgesia, as described by Sasaki and co-authors [7], although frequent antiplatelet or anticoagulant therapy in Fontan patients may limit its applicability. Thoracic epidural-induced hypotension also warrants particular vigilance, especially in patients with Fontan physiology. Another team performed an intercostal nerve block [9]. Other thoracic regional anaesthesia techniques could probably be offered.

Temperature Management

Maintenance of normothermia with fluid warmers and heating blankets is important to avoid a hypothermia-related increase in PVR.

Inotropic Support

There is no consensus regarding the optimal positive inotropic drug for Fontan patients. However, multiple publications argue in favour of milrinone, a phosphodiesterase 3 inhibitor, as the agent of choice in Fontan patients [21-24,30-32]. Hibino and colleagues administered milrinone [10]. Besides its positive inotropic effects, it also has a potent pulmonary and systemic vasodilatory action. The addition of a vasoconstrictor, e.g. vasopressin, to counteract the vasodilatory effects may be required [24].

Dobutamine, a synthetic β-agonist that decreases PVR, was previously used by three other teams [8-10] and could be used as an alternative to milrinone. In our case, we selected dobutamine for inotropic therapy, as we had already initiated a vasopressin infusion and wished to avoid exacerbating systemic vasodilatation with milrinone, which is longer acting and harder to titrate.

Levosimendan, a calcium sensitiser of myocardial troponin C, may also be considered. It is a positive inotropic and pulmonary vasodilator drug, but like milrinone, it might cause challenging systemic hypotension [31].

Dopamine, used in three of the eight cases [4,6,10], has a positive inotropic effect and acts as a systemic vascular vasoconstrictor. An increase in PVR can be observed at high doses, and it is more arrhythmogenic compared to both dobutamine and milrinone [31]. Dopamine may therefore be a less attractive choice for inotropic support in this patient group.

Arrhythmia Management

Many Fontan patients have a pacemaker, with epicardial pacing systems preferred over transvenous leads because of the anatomy and increased thromboembolic risk. For pacemaker-dependent patients, preoperative reprogramming of the device to an asynchronous, slightly higher heart rate may be desirable [31]. Our patient’s device was programmed to DOO to avoid any diathermy-induced arrhythmias, as per hospital protocol. In our case, VF occurred due to direct surgical diathermy of the ventricle and not due to an inadvertent R-on-T phenomenon caused by asynchronous pacing. Fibrillation was terminated after four external DC shocks and amiodarone administration.

Supraventricular rhythm disturbances are frequent in Fontan patients, with up to 45% having sinus node dysfunction. Prolongation of atrial refractory periods and intra-atrial conduction delays predispose to atrial tachyarrhythmias [12].

Intraoperative loss of atrioventricular synchrony will cause increased atrial pressures, reducing the Fontan gradient through the lungs and resulting in a fall in cardiac output. Preoperative correction of electrolytes (especially potassium and magnesium) will decrease the likelihood of intraoperative arrhythmias. Application of external defibrillator pads is advised, as adhesions from prior surgery may prevent effective internal defibrillator paddle placement.

ECMO as Rescue

Despite poor reported outcomes in single-ventricle physiology, veno-arterial extracorporeal membrane oxygenation (VA-ECMO) should be considered as rescue therapy to assist with circulation and oxygenation. Double venous cannulation is needed to optimise distal superior and inferior vena cava drainage [32]. Indeed, venous drainage would not be complete with only inferior caval cannulation. In our case, we decided that, in an emergency situation (profound hypotension and/or hypoxemia), femoral venous and arterial cannulation providing partial bypass would be instituted, with subsequent cannulation of one (or potentially two, due to the bilateral cavopulmonary connections) additional upper body cannula to achieve full cardiopulmonary bypass.

None of the previously published cases have reported the need for intraoperative ECMO when initiating OLV in Fontan patients.

Teamwork and Communication

Effective teamwork in the operating theatre is essential to ensure that OLV is performed under the safest possible conditions in a Fontan patient. This requires clear communication and close collaboration among nurses, surgeons, perfusionists, and anaesthetists, all of whom must be familiar with working as a coordinated team. Simulation-based training, complemented by structured debriefing sessions, can be particularly valuable in enhancing interprofessional communication, team performance, and preparedness for crises.

A pre-incision agreement with the surgical team should be established to revert to two-lung ventilation if the patient does not tolerate OLV. The possibility of alternating between two-lung ventilation and OLV should also be discussed in advance.

Postoperative care

As OLV adds an additional perioperative risk of cardiac decompensation in Fontan patients, we strongly recommend monitoring the patient postoperatively in an intensive care unit (ICU) setting. Half of the adverse events after noncardiac surgery in this patient population are related to postoperative issues [33]. Bleeding, fever, thromboembolism, infection, and pulmonary oedema are poorly tolerated. Volume management can be complex, with patients needing either additional fluid or diuresis [22]. Volume status should be assessed with a thorough and appropriately adapted clinical examination, along with advanced monitoring, such as echocardiography and CVP monitoring.

Given the risk of hypoxemia, arrhythmia, and circulatory decompensation, postoperative monitoring should include oxygen saturation, ECG and IBP monitoring. Renal and hepatic function should be closely monitored [12]. The duration of postoperative ICU care should be adjusted according to the patient's overall clinical status and the extent and indication of the surgical procedure.

Effective pain management is essential to normalise breathing patterns while avoiding significant respiratory depression, as described above. Multimodal analgesia and thoracic regional anaesthesia techniques should be proposed.

Antiemetic prophylaxis will ease the return to enteral intake, thereby permitting the patient to regulate fluid intake and restart oral medications [21].

Preoperative anticoagulation and antiplatelet therapy should be resumed as early as clinically appropriate, based on bleeding risk, to prevent thrombotic complications. Unfractionated heparin offers the advantage of rapid reversibility in the event of bleeding. Early ambulation and the use of pneumatic compression devices can further help reduce the risk of venous thromboembolism.

## Conclusions

The management of one-lung ventilation in patients with Fontan circulation represents a rare but significant anaesthetic challenge requiring a deep understanding of their unique physiology and potential vulnerabilities. Optimising preload, maintaining low pulmonary vascular resistance and anticipating perioperative complications are key to successful outcomes. Our case adds to the limited existing literature on this high-risk scenario and reinforces the importance of careful multidisciplinary planning, vigilant intraoperative monitoring and specialised postoperative care. While no standardised protocol exists, synthesis of the available reports highlights consistent principles that can help guide anaesthetists facing similar situations. Further data collection and collaborative reporting are essential to refine best practices and improve safety for this growing adult congenital heart disease population. Prospective studies are needed to better define perioperative management protocols for OLV in Fontan physiology, particularly regarding the impact of ventilatory settings and anaesthetic drugs on PVR and CO in these patients.

## References

[1] Fontan F, Baudet E (1971). Surgical repair of tricuspid atresia. Thorax.

[2] Schilling C, Dalziel K, Nunn R (2016). The Fontan epidemic: population projections from the Australia and New Zealand Fontan Registry. Int J Cardiol.

[3] Neethling E, Heggie JE (2022). Considerations in critical-care and anesthetic management of adult patients living with Fontan circulation. Can J Cardiol.

[4] Majima N, Kagawa T, Suzuki T, Kurosaki A (2012). Successful one-lung ventilation in a patient with the Fontan circulation undergoing thoracotomy: a case report. J Anesth.

[5] Cvetkovic D, Ramzy W, Vitale S, Malekan R, Warsy I (2014). Successful one-lung ventilation in a patient with the Fontan circulation undergoing thoracoscopic procedure. Semin Cardiothorac Vasc Anesth.

[6] Gregory SH, Swaminathan M, Maisonave Y, Machovec KA (2016). Management of 1-lung ventilation in a patient with failing Fontan circulation. A A Case Rep.

[7] Sasaki Y, Kato J, Minoshima R, Nagata H, Minamishima S, Suzuki T, Morisaki H (2019). One-lung ventilation in a patient with Fontan circulation undergoing diaphragmatic plication surgery: a case report. JA Clin Rep.

[8] Higashi A, Kinoshita M, Sudo K, Ueno H, Sawa T (2024). Anesthetic management of thoracoscopic pulmonary cystectomy in a patient with Fontan circulation and disturbed lung inflation during leakage testing: a case report. Cureus.

[9] Kawakami M, Ito H, Takazawa T (2024). Safe anesthetic management of one-lung ventilation in an adolescent patient with Fontan circulation: a case report. Cureus.

[10] Hibino T, Okui Y, Toba Y (2024). Anesthesia management for epicardial pacemaker electrode implantation in a patient with a history of Fontan procedure: a case report. Cureus.

[11] Haight PJ, Stewart RE, Saarel EV, Pettersson GB, Najm HK, Aziz PF (2018). Lateral thoracotomy for epicardial pacemaker placement in patients with congenital heart disease. Interact Cardiovasc Thorac Surg.

[12] Stout KK, Daniels CJ, Aboulhosn JA (2019). 2018 AHA/ACC guideline for the management of adults with congenital heart disease: executive summary: a report of the American College of Cardiology/American Heart Association Task Force on clinical practice guidelines. Circulation.

[13] Williams DB, Kiernan PD, Metke MP, Marsh HM, Danielson GK (1984). Hemodynamic response to positive end-expiratory pressure following right atrium-pulmonary artery bypass (Fontan procedure). J Thorac Cardiovasc Surg.

[14] Talbot NP, Balanos GM, Dorrington KL, Robbins PA (2005). Two temporal components within the human pulmonary vascular response to approximately 2 h of isocapnic hypoxia. J Appl Physiol (1985).

[15] Kiely DG, Cargill RI, Lipworth BJ (1996). Effects of hypercapnia on hemodynamic, inotropic, lusitropic, and electrophysiologic indices in humans. Chest.

[16] (2017). Practice guidelines for preoperative fasting and the use of pharmacologic agents to reduce the risk of pulmonary aspiration: application to healthy patients undergoing elective procedures. An updated report by the American Society of Anesthesiologists Task Force on Preoperative Fasting and the use of pharmacologic agents to reduce the risk of pulmonary aspiration. Anesthesiology.

[17] Beck DH, Doepfmer UR, Sinemus C, Bloch A, Schenk MR, Kox WJ (2001). Effects of sevoflurane and propofol on pulmonary shunt fraction during one-lung ventilation for thoracic surgery. Br J Anaesth.

[18] Pruszkowski O, Dalibon N, Moutafis M, Jugan E, Law-Koune JD, Laloë PA, Fischler M (2007). Effects of propofol vs sevoflurane on arterial oxygenation during one-lung ventilation. Br J Anaesth.

[19] Hahm TS, Jeong H, Ahn HJ (2019). Systemic oxygen delivery during one-lung ventilation: comparison between propofol and sevoflurane anaesthesia in a randomised controlled trial. J Clin Med.

[20] Wang JY, Russell GN, Page RD, Jackson M, Pennefather SH (1998). Comparison of the effects of sevoflurane and isoflurane on arterial oxygenation during one lung ventilation. Br J Anaesth.

[21] Eagle SS, Daves SM (2011). The adult with Fontan physiology: systematic approach to perioperative management for noncardiac surgery. J Cardiothorac Vasc Anesth.

[22] Leyvi G, Wasnick JD (2010). Single-ventricle patient: pathophysiology and anesthetic management. J Cardiothorac Vasc Anesth.

[23] McNamara JR, McMahon A, Griffin M (2022). Perioperative management of the Fontan patient for cardiac and noncardiac surgery. J Cardiothorac Vasc Anesth.

[24] Windsor J, Townsley MM, Briston D, Villablanca PA, Alegria JR, Ramakrishna H (2017). Fontan palliation for single-ventricle physiology: perioperative management for noncardiac surgery and analysis of outcomes. J Cardiothorac Vasc Anesth.

[25] Miller O I, Tang S F, Keech A, Celermajer DS (1995). Rebound pulmonary hypertension on withdrawal from inhaled nitric oxide. Lancet.

[26] Currigan DA, Hughes RJ, Wright CE, Angus JA, Soeding PF (2014). Vasoconstrictor responses to vasopressor agents in human pulmonary and radial arteries: an in vitro study. Anesthesiology.

[27] Bigelow AM, Ghanayem NS, Thompson NE (2019). Safety and efficacy of vasopressin after Fontan completion: a randomized pilot study. Ann Thorac Surg.

[28] Loomba RS, Gray SB, Flores S (2018). Hemodynamic effects of ketamine in children with congenital heart disease and/or pulmonary hypertension. Congenit Heart Dis.

[29] Giles TD, Iteld BJ, Mautner RK, Rognoni PA, Dillenkoffer RL (1981). Short-term effects of intravenous clonidine in congestive heart failure. Clin Pharmacol Ther.

[30] Cai J, Su Z, Shi Z, Zhou Y, Xu Z, Xu Z, Yang Y (2008). Nitric oxide and milrinone: combined effect on pulmonary circulation after Fontan-type procedure: a prospective, randomized study. Ann Thorac Surg.

[31] Roeleveld PP, de Klerk JC (2018). The perspective of the intensivist on inotropes and postoperative care following pediatric heart surgery: an international survey and systematic review of the literature. World J Pediatr Congenit Heart Surg.

[32] Zwischenberger JB, Breetz KA, Ballard-Croft C, Wang D (2022). Failing Fontan cardiovascular support: review. J Card Surg.

[33] Maxwell BG, Posner KL, Wong JK, Oakes DA, Kelly NE, Domino KB, Ramamoorthy C (2015). Factors contributing to adverse perioperative events in adults with congenital heart disease: a structured analysis of cases from the closed claims project. Congenit Heart Dis.

